# Recent advances in superwetting anti-fog surfaces for medical goggles: a mini review

**DOI:** 10.3389/fbioe.2026.1865199

**Published:** 2026-07-09

**Authors:** Qiuxu Guo, Jing Hu, Yongfeng Wang, Lei Wang

**Affiliations:** Pediatric Outpatient Department, The First Hospital of Jilin University, Changchun, China

**Keywords:** anti-fogging, medical goggles, multifunctional surfaces, superhydrophilic surfaces, superhydrophobic surfaces

## Abstract

Medical goggles often suffer from fogging and droplet adhesion in high-humidity or long-term use, severely impairing visual clarity. Superwetting anti-fog surfaces have gained attention, primarily focusing on superhydrophilic and superhydrophobic surfaces. This mini review systematically discusses the mechanism and performance of these approaches, with particular emphasis on their applications in medical goggles. In particular, it highlights the advantages, limitations, and application challenges of these two strategies in medical environments, offering insights that are often dispersed across the existing literature. Furthermore, this review proposes future development directions toward multifunctional anti-fog goggles integrating antibacterial, anti-reflective, self-healing, and self-cleaning functionalities. Such integrated designs are expected to enable more durable, reliable, and sustainable optical protection for healthcare applications.

## 1 Introduction

During the COVID-19 pandemic and routine medical practice, healthcare workers are required to wear goggles for prolonged periods in humid and enclosed environments (Jose et al., 2021). Water vapor from respiration, body fluids, and ambient moisture readily condenses on goggle surfaces, forming droplets that degrade visual clarity, reduce operational accuracy, and increase safety risks (Ye et al., 2022). Consequently, goggle fogging remains a persistent challenge in medical and protective applications (Madan et al., 2020; Li et al., 2024).

Traditional anti-fog technologies primarily rely on chemical anti-fog agents or surfactant-based treatments that reduce water surface tension and promote the formation of a continuous water film (Durán and Laroche, 2019). However, these agents are typically attached through weak physical adsorption and are therefore prone to degradation and removal during repeated cleaning, wiping, and prolonged use. In medical settings, repeated alcohol-based disinfection can dissolve or leach surface-active components, while ultraviolet sterilization may induce photochemical degradation of organic functional groups. In addition, frequent friction wiping can physically remove the anti-fog layer, and alternating temperature and humidity conditions may generate thermal stress and interfacial delamination, leading to a gradual loss of anti-fog performance. Although active anti-fog approaches such as electrically heated systems can effectively suppress condensation (Traiwattanapong et al., 2024), their reliance on continuous power input increases energy consumption and system complexity. Consequently, conventional anti-fog technologies often fail to provide durable and reliable anti-fog performance under long-term medical use and repeated sterilization conditions.

By tailoring surface micro/nanostructures and wettability, anti-fog surfaces can regulate water condensation and spreading, thereby preserving optical transparency under humid and prolonged-use conditions (Dou et al., 2024). Current anti-fog strategies for medical goggles mainly fall into two categories: superhydrophilic surfaces, which spread condensed droplets into continuous water films to prevent fog formation (Deng et al., 2023), and superhydrophobic surfaces, which combine hierarchical structures with low surface energy to induce a Cassie–Baxter state, enabling rapid droplet removal (Parvate et al., 2020; Sun et al., 2026).

Although the aforementioned anti-fog strategies have made significant progress in experimental research, medical goggles still face multiple challenges under prolonged use, including maintaining stable anti-fogging performance, preserving functionality after repeated disinfection, and achieving high optical clarity. In this context, this mini review systematically examines the mechanisms and performance of superhydrophilic and superhydrophobic anti-fog strategies, with a particular focus on their current applications in medical goggles. Furthermore, it highlights future directions for achieving comprehensive performance including anti-fogging, antibacterial, anti-reflective, self-healing, and self-cleaning properties through multi-functional integrated designs. This work provides valuable insights for the optimized design and practical engineering of optical protective equipment in both current and future medical protection scenarios.

## 2 Superwetting anti-fog surfaces and their mechanisms

In humid environments with large temperature gradients, condensation-induced fogging degrades the transparency and safety of optical devices (Crebolder and Sloan, 2004) Wettability-engineered anti-fog surfaces mitigate light scattering by regulating the state and distribution of condensed water (Wahab et al., 2023), and are generally classified as superhydrophilic or superhydrophobic. Superhydrophilic surfaces form continuous water films, whereas superhydrophobic surfaces prevent fogging by rapidly removing condensed droplets.

### 2.1 Superhydrophilic anti-fog surfaces

**FIGURE 1:**
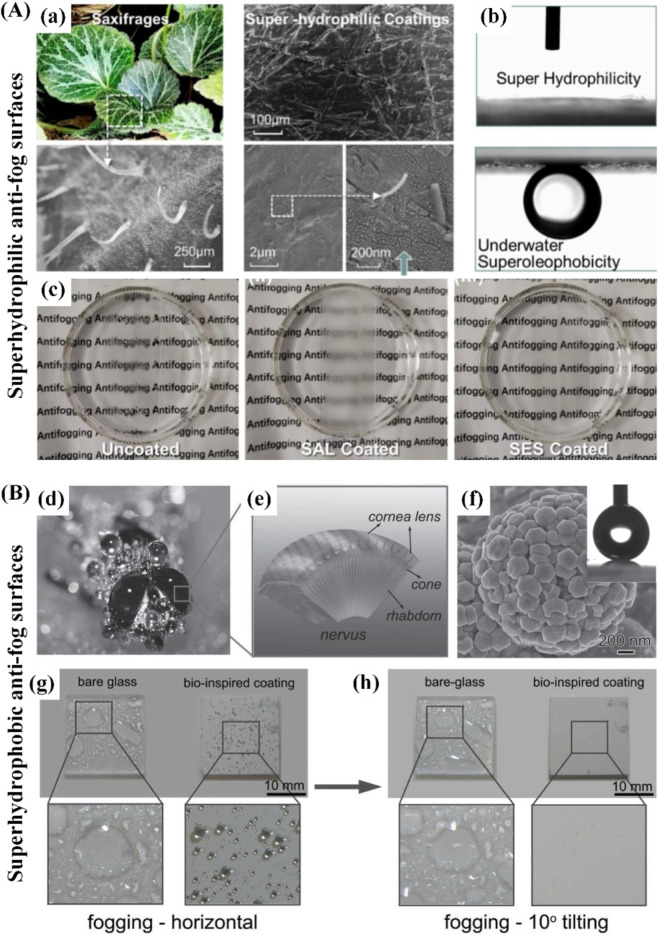
Representative superhydrophilic and superhydrophobic anti-fogging surfaces and their structure–performance relationships. **(A)** Superhydrophilic anti-fog surfaces: **(a)** Surface structures of Saxifraga and the biomimetic superhydrophilic coating (SES); **(b)** excellent superhydrophilicity of the SES coating, which promotes rapid water spreading and continuous water-film formation, **(c)** superior anti-fogging performance of SES-coated glass compared with uncoated and SAL-coated glass (waterborne styrene-acrylate emulsion) under hot-water conditions. **(B)** Superhydrophobic anti-fog surfaces: **(d)** Optical image of the compound eye of *Lucilia sericata* under foggy conditions; **(e)** schematic of the fly compound eye structure; **(f)** bio-inspired ZnO nanostructure exhibiting superhydrophobicity with high water contact angle; **(g)** anti-fogging performance after 2 min exposure in an artificial fog chamber; **(h)** droplet removal on the coating at a tilt angle of 10°, demonstrating the anti-fog mechanism based on droplet repellence and self-cleaning behavior. (Reproduced with permission from refs (Xu et al., 2025; Sun et al., 2014). Copyright 2025 Elsevier B.V.; 2014 Wiley-VCH).

Superhydrophilic surfaces with very low contact angles (<10°) enable the rapid spreading of condensate water and the formation of a continuous water film, which is superior to the discrete water droplet structure on ordinary hydrophilic surfaces. Discrete water droplets introduce a large amount of air-water interface, resulting in significant refractive index mismatch and intense light scattering (especially Mie scattering), leading to the phenomenon of fogging. In contrast, the continuous water film can significantly reduce the number of interfaces, make the refractive index distribution more uniform and smoother, lower the light scattering intensity, and improve the light transmittance (Deng et al., 2023; Cebeci et al., 2006). Therefore, from the perspective of optical propagation, the continuous water film can more effectively inhibit fogging. Xu et al. (2025) reported a Saxifraga-inspired cellulose-based superhydrophilic coating with a micro/nano hierarchical structure formed via a one-step semi-wet esterification process, achieving a water contact angle of 0° (Figures 1a,b). Mimicking the hierarchical surface architecture of Saxifraga, the coating provides abundant hydrophilic sites and capillary channels that promote rapid water spreading and the formation of a continuous thin water film, thereby preventing the accumulation of discrete light-scattering droplets. As a result, the coating exhibited excellent anti-fogging performance under humid conditions (Figure 1c) while maintaining stable superhydrophilicity after 600 abrasion cycles. In addition, the coating offers the advantages of environmental friendliness, simple fabrication, and low cost.

### 2.2 Superhydrophobic anti-fog surfaces

Superhydrophobic surfaces are created by integrating low-surface-energy materials with micro-/nano-scale hierarchical structures, resulting in a Cassie–Baxter wetting state that minimizes the solid–liquid contact area. As a result, condensed water droplets have limited adhesion to the surface and readily roll off or detach, thereby maintaining surface dryness and optical transparency. Sun et al. (2014) fabricated fly-eye-inspired inorganic nanostructures via a two-step self-assembly process (Figures 1d–f). Mimicking the hierarchical architecture of fly compound eyes, the nanostructured surface effectively minimizes the solid–liquid contact area and reduces droplet adhesion, resulting in ultralow contact angle hysteresis. Consequently, condensed droplets can rapidly roll off the surface before growing into light-scattering fog droplets, leading to excellent anti-fogging performance under humid conditions (Figures 1g,h). This biomimetic strategy provides valuable design guidance for anti-fog materials operating under extreme environments. Lecointre et al. (2021) further demonstrated that nanocone arrays significantly enhance microdroplet repellency compared with columnar nanostructures. The sharp nanocone geometry maintains condensed microdroplets in a highly non-adhesive state, enabling droplets with radii as small as 1.5 μm to detach rapidly from the surface and thereby sustain stable anti-fogging behavior. Moreover, truncated nanocone structures facilitated efficient liquid drainage through repeated droplet coalescence, thereby broadening the structural design space for high-performance anti-fog surfaces.

Overall, superhydrophilic surfaces promote the rapid spreading of condensed water into a continuous thin film owing to their extremely low contact angles. This effectively suppresses the formation of fog droplets, reduces light scattering, and maintains optical stability and transparency, thereby realizing an anti-fogging mechanism centered on regulating the state of condensed water. In contrast, superhydrophobic surfaces achieve self-cleaning anti-fogging through the construction of micro-/nano-scale structures with low surface energy, which inhibit droplet adhesion and enable rapid droplet removal. This represents an alternative anti-fogging strategy based on droplet removal and provides an important theoretical foundation and practical guidance for the design of anti-fog optical devices, such as goggles.

## 3 Application prospects of anti-fog surfaces in medical goggles

**FIGURE 2:**
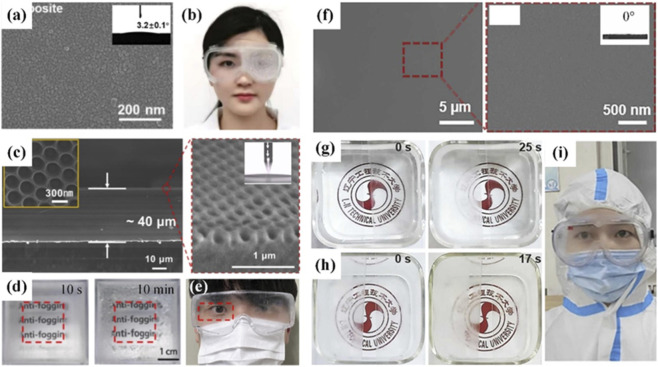
Typical anti-fogging coatings for medical optical applications and their performance under different environmental conditions. **(a,b)** Composite coating: **(a)** SEM images and water contact angle; **(b)** comparison of anti-fogging performance between composite-coated (left) and bare (right) lenses after 30 min exposure to water vapor above 80 °C, demonstrating enhanced anti-fogging property. **(c–e)** PBPS coating: **(c)** Cross-sectional SEM image of PBPS with nanostructures; **(d)** anti-fogging performance under hot water vapor; **(e)** fogging resistance test of PBPS-coated goggles. **(f–i)** Transparent superhydrophilic coating: **(f)** Surface morphology and low contact angle; **(g)** snapshots above a 55 °C heat source and **(h)** after −18 °C storage for 1 h followed by room-temperature exposure; **(i)** clear vision maintained on coated goggles, indicating the combined advantages of transparency and rapid water spreading. (Reproduced with permission from refs (Deng et al., 2023; Kim et al., 2025; Yang et al., 2023). Copyright 2023 Elsevier Inc.; 2025 Elsevier B.V.; 2023 Elsevier Ltd.).

With the widespread use of medical goggles, durable and reliable anti-fog performance under prolonged high-humidity conditions has become increasingly critical (Keschner et al., 2023). Wettability-engineered anti-fog surfaces effectively regulate water condensation behavior, thereby preserving optical clarity and improving medical safety (Li et al., 2025). Recently, superhydrophilic anti-fog coatings have shown strong potential for medical goggles. Deng et al. (2023) developed a ceramic–polymer composite coating with an ultralow water contact angle (Figure 2a), high visible-light transmittance (91.8%), and excellent durability, maintaining stable anti-fogging performance after severe abrasion (under 400 continuous cycles of abrasion at 250 g of load) and boiling-water tests (immersion in boiling water for 30 min) and demonstrating clear vision in practical goggle applications (Figure 2b). Kim et al. (2025) developed a polymer brush–type perfluoropolyether film (PBPS) integrating moth-eye nanostructures and hydrophilic polymer brushes, enabling simultaneous anti-reflection and superhydrophilic anti-fogging with transmittance exceeding 97% under dry conditions (26 °C, 30% RH) and 95% under wet conditions (49 °C, lid surface temperature 45 °C, 100% RH), and maintaining clear vision in goggle tests under high humidity (Figures 2c–e). Yang et al. (2023) reported a transparent multifunctional superhydrophilic coating prepared via co-deposition and biomimetic mineralization, exhibiting near-zero water contact angle, and excellent anti-fogging performance in practical goggle applications (Figures 2f–i).

In addition to superhydrophilic anti-fogging strategies, superhydrophobic surfaces have also attracted increasing attention in the field of anti-fog due to its unique advantages in suppressing droplet adhesion, enabling rapid water removal, and imparting self-cleaning functionality (Kong et al., 2024). Although the systematic application of superhydrophobic surfaces in medical goggles remains relatively limited at present, encouraging progress has been reported in transparent optical devices such as eyeglasses. For example, Jang et al. (2019) developed a simple, rapid, and low-cost approach to fabricate highly flexible and transparent superhydrophobic polymer films. These films combine high optical transmittance with excellent mechanical flexibility while maintaining stable superhydrophobicity, and they demonstrated effective anti-fogging performance in eyeglass fogging tests, highlighting their potential for application in transparent optical protective devices, including goggles. Furthermore, the large-scale application of superhydrophobic coatings on medical goggles still faces several critical bottlenecks, as summarized below. Achieving superhydrophobicity often requires micro/nanostructures that increase light scattering and reduce optical transmittance. In addition, the fragile surface structures are susceptible to mechanical abrasion during daily use and cleaning. Repeated exposure to disinfectants may also degrade the coating chemistry and surface morphology, resulting in a loss of water repellency. Moreover, the coating materials must exhibit excellent biocompatibility and safety for long-term medical use. Therefore, balancing transparency, wettability, mechanical durability, chemical stability, and biocompatibility remains a major challenge.

In summary, anti-fogging strategies based on surface wettability regulation have shown promising application prospects in medical goggles. Superhydrophilic coatings achieve stable and effective anti-fogging by promoting the rapid spreading of condensed water into a continuous water film, with their performance validated in terms of optical transmittance, durability, and practical goggle tests. Meanwhile, superhydrophobic surfaces rely on low surface energy and hierarchical micro/nanostructures to facilitate rapid droplet detachment and self-cleaning, and have also demonstrated favorable anti-fogging performance in transparent optical devices. Together, these studies indicate that anti-fog surfaces with distinct wettability mechanisms offer diverse design concepts and technical pathways for medical goggle anti-fogging, laying a solid foundation for the development of high-performance and multifunctional anti-fog goggles.

## 4 Conclusion and outlook

This review systematically summarizes recent advances in superwetting anti-fog surfaces, with particular emphasis on superhydrophilic and superhydrophobic coatings for medical optical devices such as goggles. The anti-fogging mechanisms, structural characteristics, and practical performance of different coating systems are discussed and compared.

Although significant progress has been achieved, several challenges still hinder the large-scale application of anti-fog coatings in medical environments. For multifunctional integration, combining anti-fogging properties with antibacterial, anti-reflective, self-cleaning, and self-healing functions often leads to interfacial compatibility issues and performance trade-offs. For example, surface roughness required for superhydrophobicity may increase optical scattering, while antibacterial additives may affect coating transparency and wettability. Future studies should therefore focus on rational interface engineering and hierarchical structural design to achieve synergistic rather than competing functionalities.

Regarding durability, future research should prioritize resistance to mechanical abrasion, repeated disinfection, and environmental aging. Since medical goggles are frequently subjected to wiping, sterilization with alcohol- or chlorine-based disinfectants, and prolonged exposure to humid environments, developing robust coating-substrate adhesion, chemically stable low-surface-energy materials, and self-reinforcing micro/nanostructures will be essential for maintaining long-term anti-fog performance.

In addition, emerging technologies provide new opportunities for intelligent anti-fog surfaces. Stimuli-responsive wetting surfaces capable of dynamically switching wettability in response to temperature, humidity, light, or pH may enable adaptive anti-fogging under varying operating conditions. Amphiphilic composite structures that integrate hydrophilic water-spreading domains with hydrophobic water-repellent regions also represent a promising strategy for simultaneously improving condensation management, optical transparency, and environmental adaptability.

In summary, future research should move beyond conventional single-function anti-fog coatings toward multifunctional, durable, and intelligent systems specifically designed for medical application scenarios. Addressing the challenges of functional compatibility, long-term durability under repeated sterilization, and adaptive wetting regulation will be critical for the practical deployment of next-generation anti-fog medical optical protective equipment.
